# [(*Z*)-*O*-Ethyl-*N*-propyl­thio­carbamato-κ*S*](triphenyl­phosphine-κ*P*)gold(I)

**DOI:** 10.1107/S1600536809047254

**Published:** 2009-11-14

**Authors:** Primjira P. Tadbuppa, Edward R. T. Tiekink

**Affiliations:** a Department of Chemistry, National University of Singapore, Singapore 117543; b Department of Chemistry, University of Malaya, 50603 Kuala Lumpur, Malaysia

## Abstract

The title compound, [Au(C_6_H_12_NOS)(C_18_H_15_P)], features a linear *S*,*P*-donor set about the central Au atom with a deviation from linearity [S—Au—P = 176.66 (5)°] due to an intra­molecular Au⋯O contact [2.991 (5) Å]. Supra­molecular dimers are formed in the crystal structure mediated by C—H⋯N inter­actions.

## Related literature

For structural systematics and luminescence properties of phosphinegold(I) carbonimidothio­ates, see: Ho *et al.* (2006 ▶); Ho & Tiekink (2007 ▶); Kuan *et al.* (2008 ▶). For the synthesis, see: Hall *et al.* (1993 ▶). For a description of phenyl embraces, see: Dance & Scudder (2009 ▶).
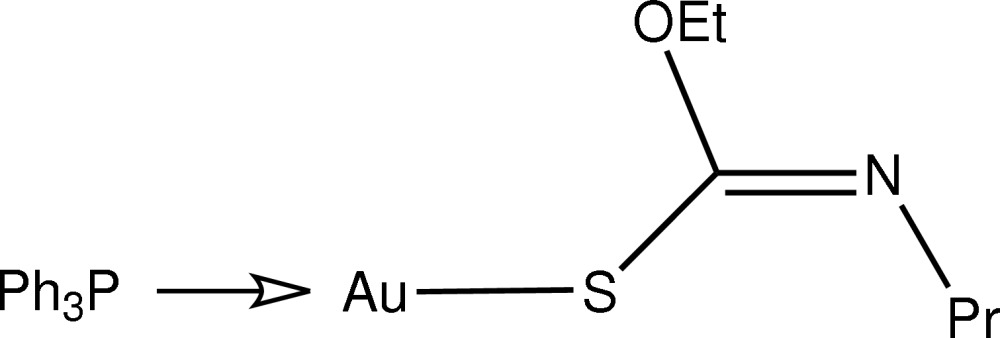


## Experimental

### 

#### Crystal data

[Au(C_6_H_12_NOS)(C_18_H_15_P)]
*M* *_r_* = 605.46
Monoclinic, 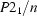

*a* = 8.5822 (6) Å
*b* = 18.2036 (13) Å
*c* = 15.6893 (12) Å
β = 102.001 (2)°
*V* = 2397.5 (3) Å^3^
*Z* = 4
Mo *K*α radiation
μ = 6.30 mm^−1^
*T* = 223 K
0.16 × 0.05 × 0.04 mm

#### Data collection

Bruker SMART CCD diffractometer
Absorption correction: multi-scan (*SADABS*, Bruker, 2000 ▶) *T*_min_ = 0.439, *T*_max_ = 1
16839 measured reflections
5496 independent reflections
4350 reflections with *I* > 2σ(*I*)
*R*_int_ = 0.042

#### Refinement

*R*[*F* ^2^ > 2σ(*F* ^2^)] = 0.035
*wR*(*F* ^2^) = 0.098
*S* = 1.07
5496 reflections
262 parameters
H-atom parameters constrained
Δρ_max_ = 1.19 e Å^−3^
Δρ_min_ = −0.63 e Å^−3^

### 

Data collection: *SMART* (Bruker, 2000 ▶); cell refinement: *SAINT* (Bruker, 2000 ▶); data reduction: *SAINT*; program(s) used to solve structure: *PATTY* in *DIRDIF92* (Beurskens *et al.*, 1992 ▶); program(s) used to refine structure: *SHELXL97* (Sheldrick, 2008 ▶); molecular graphics: *DIAMOND* (Brandenburg, 2006 ▶); software used to prepare material for publication: *publCIF* (Westrip, 2009 ▶).

## Supplementary Material

Crystal structure: contains datablocks global, I. DOI: 10.1107/S1600536809047254/hg2586sup1.cif

Structure factors: contains datablocks I. DOI: 10.1107/S1600536809047254/hg2586Isup2.hkl

Additional supplementary materials:  crystallographic information; 3D view; checkCIF report

## Figures and Tables

**Table 1:** Selected bond lengths (Å)

Au—S1	2.2980 (14)
Au—P1	2.2561 (14)

**Table 2:** Hydrogen-bond geometry (Å, °)

*D*—H⋯*A*	*D*—H	H⋯*A*	*D*⋯*A*	*D*—H⋯*A*
C8—H8⋯N1^i^	0.94	2.54	3.433 (9)	159

Symmetry code: (i) 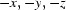.

**Fig. 1.**
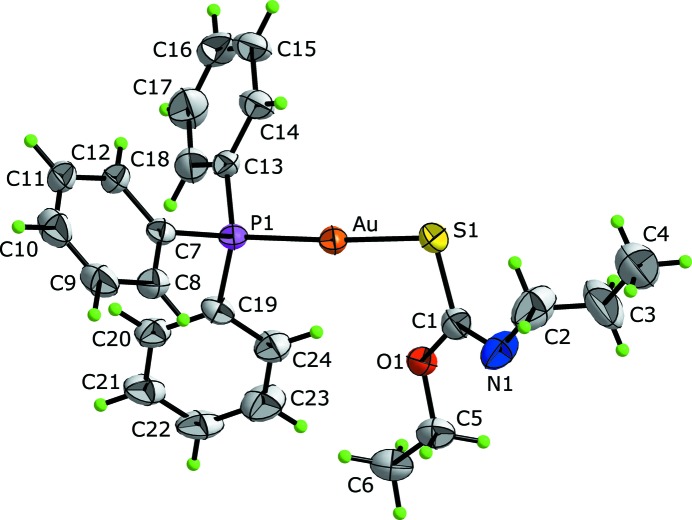
Molecular structure of (I) showing atom-labelling scheme and displacement ellipsoids at the 50% probability level.

**Fig. 2.**
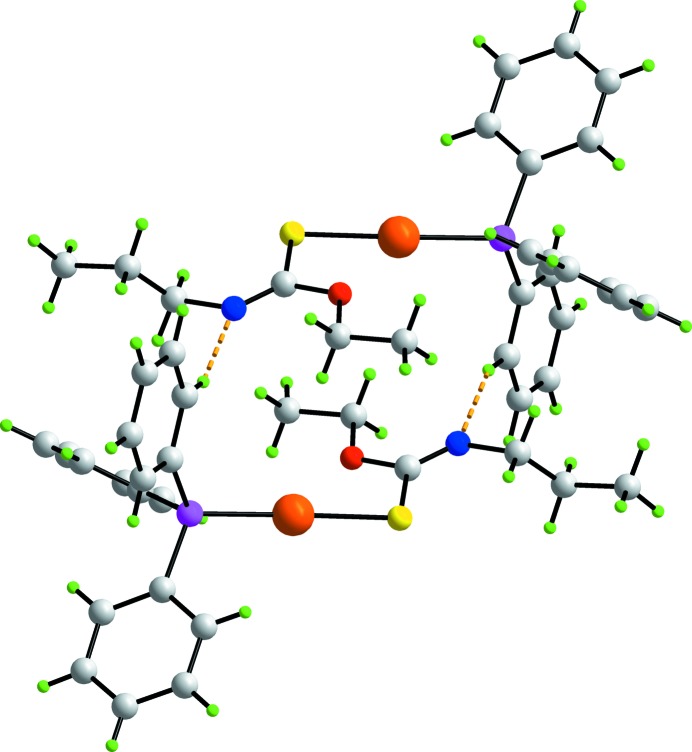
Supramolecular dimer in (I) mediated by C—H···N contacts (orange dashed lines). Colour code: Au, orange; S, yellow; P, pink; O, red; N, blue; C, grey; and H, green.

## supplementary crystallographic information

### Comment

As a part of systematic studies of phosphinegold(I) thiocarbamides (Ho *et al.* 2006; Ho & Tiekink, 2007; Kuan *et al.*, 2008), the title compound, Ph_3_Au[SC(OEt)NC_3_H_7_], was synthesized.

The gold atom exists in the expected linear geometry defined by a SP donor set, Table 1 and Fig. 1, and the deviation from linearity [S1–Au–P1 is 176.66 (5) Å] is traced to the close approach of the O1 atom to Au [Au···O = 2.991 (5) Å]. The anion, with C1—S1 and C1—N1 bond distances of 1.765 (7) and 1.262 (8)Å, respectively, coordinates as a thiolate; the configuration about the double bond is *Z*.

In the crystal structure of (I), supramolecular dimers are formed owing to the presence of C–H···N interactions, Table 2 and Fig. 2. Dimers are connected by sixfold phenyl embraces with a P1···P1^i^ separation of 6.836 (2) Å for i: -*x*, -*y*, 1 - *z* (Dance & Scudder, 2009). These cooperate with C–H···π interactions [C4–H4c···*Cg*^ii^ = 2.97 Å, C4···*Cg*^ii^ = 3.827 (11) Å with an angle at H4c = 148° for ii: 1/2 + *x*, 1/2 - *y*, -1/2 + *z*, where *Cg* is the ring centroid of C13—C18] to consolidate the crystal structure.

### Experimental

Compound (I) was prepared following the standard literature procedure from the reaction of Ph_3_AuCl and EtOC(S)N(H)C_3_H_7_ in the presence of base (Hall *et al.*, 1993).

### Refinement

The H atoms were geometrically placed (C—H = 0.94–0.98 Å) and refined as riding with *U*_iso_(H) = 1.2–1.5*U*_eq_(C). The maximum and minimum residual electron density peaks of 1.19 and 0.63 e Å^-3^, respectively, were located 0.92 Å and 1.31 Å from the Au and P1 atoms, respectively.

### Crystal data

**Table:**

[Au(C_6_H_12_NOS)(C_18_H_15_P)]	*F*(000) = 1184
*M**_r_* = 605.46	*D*_x_ = 1.677 Mg m^−^^3^
Monoclinic, *P*2_1_/*n*	Mo *K*α radiation, λ = 0.71069 Å
Hall symbol: -P 2yn	Cell parameters from 3440 reflections
*a* = 8.5822 (6) Å	θ = 2.3–24.6°
*b* = 18.2036 (13) Å	µ = 6.30 mm^−^^1^
*c* = 15.6893 (12) Å	*T* = 223 K
β = 102.001 (2)°	Block, colourless
*V* = 2397.5 (3) Å^3^	0.16 × 0.05 × 0.04 mm
*Z* = 4	

### Data collection

**Table:**

Bruker SMART CCD diffractometer	5496 independent reflections
Radiation source: fine-focus sealed tube	4350 reflections with *I* > 2σ(*I*)
graphite	*R*_int_ = 0.042
ω scans	θ_max_ = 27.5°, θ_min_ = 1.7°
Absorption correction: multi-scan (*SADABS*, Bruker, 2000)	*h* = −9→11
*T*_min_ = 0.439, *T*_max_ = 1	*k* = −23→20
16839 measured reflections	*l* = −19→20

### Refinement

**Table:**

Refinement on *F*^2^	Primary atom site location: structure-invariant direct methods
Least-squares matrix: full	Secondary atom site location: difference Fourier map
*R*[*F*^2^ > 2σ(*F*^2^)] = 0.035	Hydrogen site location: inferred from neighbouring sites
*wR*(*F*^2^) = 0.098	H-atom parameters constrained
*S* = 1.07	*w* = 1/[σ^2^(*F*_o_^2^) + (0.0474*P*)^2^ + 0.1712*P*] where *P* = (*F*_o_^2^ + 2*F*_c_^2^)/3
5496 reflections	(Δ/σ)_max_ < 0.001
262 parameters	Δρ_max_ = 1.19 e Å^−^^3^
0 restraints	Δρ_min_ = −0.63 e Å^−^^3^

### Special details

**Table:**

Geometry. All s.u.'s (except the s.u. in the dihedral angle between two l.s. planes) are estimated using the full covariance matrix. The cell s.u.'s are taken into account individually in the estimation of s.u.'s in distances, angles and torsion angles; correlations between s.u.'s in cell parameters are only used when they are defined by crystal symmetry. An approximate (isotropic) treatment of cell s.u.'s is used for estimating s.u.'s involving l.s. planes.
Refinement. Refinement of *F*^2^ against ALL reflections. The weighted *R*-factor *wR* and goodness of fit *S* are based on *F*^2^, conventional *R*-factors *R* are based on *F*, with *F* set to zero for negative *F*^2^. The threshold expression of *F*^2^ > 2σ(*F*^2^) is used only for calculating *R*-factors(gt) *etc*. and is not relevant to the choice of reflections for refinement. *R*-factors based on *F*^2^ are statistically about twice as large as those based on *F*, and *R*- factors based on ALL data will be even larger.

### Fractional atomic coordinates and isotropic or equivalent isotropic displacement parameters (Å^2^)

**Table:**

	*x*	*y*	*z*	*U*_iso_*/*U*_eq_	
Au	0.14256 (2)	0.109862 (11)	0.186783 (14)	0.03589 (9)	
S1	0.22776 (17)	0.15331 (8)	0.06673 (10)	0.0419 (3)	
P1	0.07358 (15)	0.06794 (7)	0.30913 (9)	0.0332 (3)	
O1	−0.0549 (5)	0.10164 (18)	0.0059 (3)	0.0400 (9)	
N1	0.0878 (7)	0.1174 (3)	−0.0998 (4)	0.0607 (15)	
C1	0.0790 (7)	0.1216 (3)	−0.0206 (4)	0.0423 (13)	
C2	0.2390 (10)	0.1360 (6)	−0.1259 (5)	0.085 (3)	
H2A	0.3195	0.1473	−0.0734	0.102*	
H2B	0.2759	0.0929	−0.1535	0.102*	
C3	0.2276 (11)	0.1954 (5)	−0.1832 (8)	0.115 (4)	
H3A	0.2024	0.2400	−0.1538	0.138*	
H3B	0.1406	0.1866	−0.2335	0.138*	
C4	0.3849 (12)	0.2072 (6)	−0.2153 (7)	0.108 (3)	
H4A	0.3752	0.2505	−0.2521	0.161*	
H4B	0.4058	0.1647	−0.2484	0.161*	
H4C	0.4720	0.2139	−0.1655	0.161*	
C5	−0.1837 (8)	0.0730 (4)	−0.0615 (4)	0.0529 (16)	
H5A	−0.1479	0.0296	−0.0889	0.063*	
H5B	−0.2187	0.1102	−0.1065	0.063*	
C6	−0.3173 (8)	0.0532 (4)	−0.0180 (5)	0.0609 (17)	
H6A	−0.4058	0.0340	−0.0610	0.091*	
H6B	−0.3514	0.0965	0.0091	0.091*	
H6C	−0.2813	0.0161	0.0262	0.091*	
C7	0.1748 (6)	−0.0157 (3)	0.3517 (4)	0.0343 (11)	
C8	0.1567 (7)	−0.0772 (3)	0.2967 (4)	0.0441 (13)	
H8	0.0916	−0.0748	0.2406	0.053*	
C9	0.2357 (8)	−0.1410 (3)	0.3259 (5)	0.0554 (17)	
H9	0.2249	−0.1823	0.2891	0.066*	
C10	0.3301 (8)	−0.1454 (3)	0.4080 (5)	0.0573 (17)	
H10	0.3822	−0.1897	0.4269	0.069*	
C11	0.3491 (7)	−0.0861 (3)	0.4627 (4)	0.0487 (15)	
H11	0.4138	−0.0893	0.5188	0.058*	
C12	0.2714 (6)	−0.0208 (3)	0.4340 (4)	0.0393 (12)	
H12	0.2846	0.0203	0.4710	0.047*	
C13	0.1185 (6)	0.1331 (3)	0.3973 (4)	0.0373 (12)	
C14	0.2545 (7)	0.1761 (3)	0.4030 (5)	0.0524 (16)	
H14	0.3186	0.1711	0.3615	0.063*	
C15	0.2949 (9)	0.2265 (3)	0.4705 (5)	0.064 (2)	
H15	0.3868	0.2556	0.4749	0.077*	
C16	0.2008 (11)	0.2338 (4)	0.5306 (5)	0.076 (2)	
H16	0.2299	0.2674	0.5766	0.091*	
C17	0.0651 (11)	0.1929 (4)	0.5246 (5)	0.072 (2)	
H17	−0.0002	0.1997	0.5652	0.086*	
C18	0.0245 (8)	0.1420 (4)	0.4592 (4)	0.0519 (15)	
H18	−0.0671	0.1130	0.4561	0.062*	
C19	−0.1353 (6)	0.0460 (3)	0.2935 (4)	0.0373 (12)	
C20	−0.1923 (7)	−0.0064 (3)	0.3443 (4)	0.0475 (14)	
H20	−0.1217	−0.0316	0.3884	0.057*	
C21	−0.3544 (8)	−0.0206 (4)	0.3289 (5)	0.0600 (18)	
H21	−0.3940	−0.0554	0.3634	0.072*	
C22	−0.4584 (7)	0.0158 (4)	0.2634 (5)	0.0630 (19)	
H22	−0.5679	0.0050	0.2529	0.076*	
C23	−0.4032 (8)	0.0672 (5)	0.2141 (5)	0.070 (2)	
H23	−0.4746	0.0927	0.1706	0.084*	
C24	−0.2415 (7)	0.0817 (4)	0.2280 (5)	0.0528 (16)	
H24	−0.2034	0.1162	0.1927	0.063*	

### Atomic displacement parameters (Å^2^)

**Table:**

	*U*^11^	*U*^22^	*U*^33^	*U*^12^	*U*^13^	*U*^23^
Au	0.03006 (12)	0.03743 (13)	0.03915 (13)	−0.00125 (9)	0.00480 (8)	0.00592 (9)
S1	0.0394 (7)	0.0456 (8)	0.0407 (8)	−0.0062 (6)	0.0085 (6)	0.0044 (6)
P1	0.0251 (6)	0.0369 (7)	0.0361 (7)	−0.0021 (5)	0.0030 (5)	0.0027 (6)
O1	0.038 (2)	0.038 (2)	0.043 (2)	−0.0036 (16)	0.0052 (18)	−0.0004 (16)
N1	0.050 (3)	0.091 (4)	0.041 (3)	0.007 (3)	0.008 (3)	0.008 (3)
C1	0.037 (3)	0.040 (3)	0.050 (4)	0.002 (2)	0.009 (3)	0.005 (2)
C2	0.068 (5)	0.139 (8)	0.052 (5)	−0.002 (5)	0.019 (4)	0.006 (5)
C3	0.076 (6)	0.097 (7)	0.168 (11)	−0.030 (5)	0.016 (7)	0.058 (7)
C4	0.134 (9)	0.095 (7)	0.095 (8)	−0.037 (6)	0.026 (7)	0.003 (6)
C5	0.055 (4)	0.049 (4)	0.053 (4)	−0.007 (3)	0.006 (3)	−0.011 (3)
C6	0.049 (4)	0.064 (4)	0.067 (5)	−0.011 (3)	0.005 (3)	−0.010 (4)
C7	0.026 (3)	0.038 (3)	0.039 (3)	−0.004 (2)	0.007 (2)	0.003 (2)
C8	0.040 (3)	0.045 (3)	0.045 (4)	−0.004 (3)	0.005 (3)	−0.002 (3)
C9	0.054 (4)	0.036 (3)	0.076 (5)	−0.003 (3)	0.012 (4)	−0.005 (3)
C10	0.049 (4)	0.040 (3)	0.082 (5)	0.005 (3)	0.012 (4)	0.012 (3)
C11	0.037 (3)	0.050 (3)	0.053 (4)	0.000 (3)	−0.004 (3)	0.018 (3)
C12	0.037 (3)	0.040 (3)	0.038 (3)	0.001 (2)	0.002 (2)	0.006 (2)
C13	0.037 (3)	0.033 (3)	0.037 (3)	−0.001 (2)	−0.004 (2)	0.003 (2)
C14	0.041 (3)	0.036 (3)	0.074 (5)	−0.004 (3)	−0.003 (3)	0.003 (3)
C15	0.057 (4)	0.041 (3)	0.084 (6)	−0.012 (3)	−0.012 (4)	−0.001 (3)
C16	0.108 (7)	0.040 (4)	0.062 (5)	0.000 (4)	−0.021 (5)	−0.011 (3)
C17	0.100 (6)	0.066 (5)	0.052 (5)	−0.004 (4)	0.023 (4)	−0.011 (4)
C18	0.059 (4)	0.048 (3)	0.048 (4)	−0.002 (3)	0.012 (3)	0.002 (3)
C19	0.028 (3)	0.039 (3)	0.047 (3)	−0.003 (2)	0.014 (2)	−0.004 (2)
C20	0.032 (3)	0.064 (4)	0.046 (4)	−0.006 (3)	0.007 (3)	0.000 (3)
C21	0.044 (4)	0.084 (5)	0.056 (4)	−0.026 (3)	0.020 (3)	−0.011 (4)
C22	0.027 (3)	0.092 (5)	0.069 (5)	−0.014 (3)	0.008 (3)	−0.016 (4)
C23	0.032 (3)	0.098 (6)	0.075 (5)	0.005 (4)	0.003 (3)	0.005 (4)
C24	0.033 (3)	0.058 (4)	0.063 (5)	0.002 (3)	0.001 (3)	0.006 (3)

### Geometric parameters (Å, °)

**Table:**

Au—S1	2.2980 (14)	C9—C10	1.374 (10)
Au—P1	2.2561 (14)	C9—H9	0.9400
S1—C1	1.765 (7)	C10—C11	1.368 (9)
P1—C13	1.802 (6)	C10—H10	0.9400
P1—C19	1.803 (5)	C11—C12	1.390 (8)
P1—C7	1.811 (5)	C11—H11	0.9400
O1—C1	1.351 (7)	C12—H12	0.9400
O1—C5	1.458 (7)	C13—C14	1.392 (8)
N1—C1	1.262 (8)	C13—C18	1.395 (8)
N1—C2	1.479 (9)	C14—C15	1.390 (9)
C2—C3	1.397 (12)	C14—H14	0.9400
C2—H2A	0.9800	C15—C16	1.369 (11)
C2—H2B	0.9800	C15—H15	0.9400
C3—C4	1.550 (12)	C16—C17	1.369 (11)
C3—H3A	0.9800	C16—H16	0.9400
C3—H3B	0.9800	C17—C18	1.372 (9)
C4—H4A	0.9700	C17—H17	0.9400
C4—H4B	0.9700	C18—H18	0.9400
C4—H4C	0.9700	C19—C24	1.385 (8)
C5—C6	1.495 (9)	C19—C20	1.395 (8)
C5—H5A	0.9800	C20—C21	1.386 (8)
C5—H5B	0.9800	C20—H20	0.9400
C6—H6A	0.9700	C21—C22	1.383 (10)
C6—H6B	0.9700	C21—H21	0.9400
C6—H6C	0.9700	C22—C23	1.360 (10)
C7—C12	1.385 (7)	C22—H22	0.9400
C7—C8	1.402 (8)	C23—C24	1.385 (9)
C8—C9	1.375 (9)	C23—H23	0.9400
C8—H8	0.9400	C24—H24	0.9400
			
P1—Au—S1	176.66 (5)	C10—C9—C8	121.0 (6)
C1—S1—Au	102.8 (2)	C10—C9—H9	119.5
C13—P1—C19	107.4 (3)	C8—C9—H9	119.5
C13—P1—C7	105.1 (2)	C11—C10—C9	120.8 (6)
C19—P1—C7	104.6 (2)	C11—C10—H10	119.6
C13—P1—Au	112.22 (18)	C9—C10—H10	119.6
C19—P1—Au	112.84 (19)	C10—C11—C12	119.0 (6)
C7—P1—Au	114.06 (17)	C10—C11—H11	120.5
C1—O1—C5	116.0 (5)	C12—C11—H11	120.5
C1—N1—C2	119.6 (6)	C7—C12—C11	120.9 (5)
N1—C1—O1	121.1 (6)	C7—C12—H12	119.6
N1—C1—S1	127.1 (5)	C11—C12—H12	119.6
O1—C1—S1	111.9 (4)	C14—C13—C18	119.2 (6)
C3—C2—N1	114.0 (8)	C14—C13—P1	117.5 (5)
C3—C2—H2A	108.7	C18—C13—P1	123.3 (5)
N1—C2—H2A	108.7	C15—C14—C13	119.6 (7)
C3—C2—H2B	108.7	C15—C14—H14	120.2
N1—C2—H2B	108.7	C13—C14—H14	120.2
H2A—C2—H2B	107.6	C16—C15—C14	119.9 (7)
C2—C3—C4	111.5 (8)	C16—C15—H15	120.0
C2—C3—H3A	109.3	C14—C15—H15	120.0
C4—C3—H3A	109.3	C15—C16—C17	121.0 (7)
C2—C3—H3B	109.3	C15—C16—H16	119.5
C4—C3—H3B	109.3	C17—C16—H16	119.5
H3A—C3—H3B	108.0	C16—C17—C18	119.9 (7)
C3—C4—H4A	109.5	C16—C17—H17	120.1
C3—C4—H4B	109.5	C18—C17—H17	120.1
H4A—C4—H4B	109.5	C17—C18—C13	120.4 (6)
C3—C4—H4C	109.5	C17—C18—H18	119.8
H4A—C4—H4C	109.5	C13—C18—H18	119.8
H4B—C4—H4C	109.5	C24—C19—C20	119.3 (5)
O1—C5—C6	107.1 (5)	C24—C19—P1	118.7 (4)
O1—C5—H5A	110.3	C20—C19—P1	122.0 (5)
C6—C5—H5A	110.3	C21—C20—C19	119.2 (6)
O1—C5—H5B	110.3	C21—C20—H20	120.4
C6—C5—H5B	110.3	C19—C20—H20	120.4
H5A—C5—H5B	108.6	C22—C21—C20	120.6 (6)
C5—C6—H6A	109.5	C22—C21—H21	119.7
C5—C6—H6B	109.5	C20—C21—H21	119.7
H6A—C6—H6B	109.5	C23—C22—C21	120.3 (6)
C5—C6—H6C	109.5	C23—C22—H22	119.8
H6A—C6—H6C	109.5	C21—C22—H22	119.8
H6B—C6—H6C	109.5	C22—C23—C24	119.9 (7)
C12—C7—C8	119.2 (5)	C22—C23—H23	120.1
C12—C7—P1	123.3 (4)	C24—C23—H23	120.1
C8—C7—P1	117.4 (4)	C23—C24—C19	120.7 (6)
C9—C8—C7	119.0 (6)	C23—C24—H24	119.6
C9—C8—H8	120.5	C19—C24—H24	119.6
C7—C8—H8	120.5		
			
P1—Au—S1—C1	159.5 (8)	C7—P1—C13—C14	89.8 (5)
S1—Au—P1—C13	57.7 (9)	Au—P1—C13—C14	−34.7 (5)
S1—Au—P1—C19	179 (100)	C19—P1—C13—C18	20.9 (6)
S1—Au—P1—C7	−61.8 (9)	C7—P1—C13—C18	−90.0 (5)
C2—N1—C1—O1	−176.8 (6)	Au—P1—C13—C18	145.5 (5)
C2—N1—C1—S1	4.1 (9)	C18—C13—C14—C15	0.6 (9)
C5—O1—C1—N1	2.2 (8)	P1—C13—C14—C15	−179.3 (5)
C5—O1—C1—S1	−178.6 (4)	C13—C14—C15—C16	−0.2 (10)
Au—S1—C1—N1	−163.6 (5)	C14—C15—C16—C17	−1.1 (11)
Au—S1—C1—O1	17.3 (4)	C15—C16—C17—C18	2.1 (12)
C1—N1—C2—C3	−118.2 (9)	C16—C17—C18—C13	−1.8 (11)
N1—C2—C3—C4	−174.3 (8)	C14—C13—C18—C17	0.4 (9)
C1—O1—C5—C6	178.9 (5)	P1—C13—C18—C17	−179.8 (5)
C13—P1—C7—C12	−3.5 (5)	C13—P1—C19—C24	98.2 (5)
C19—P1—C7—C12	−116.4 (5)	C7—P1—C19—C24	−150.5 (5)
Au—P1—C7—C12	119.9 (4)	Au—P1—C19—C24	−26.0 (5)
C13—P1—C7—C8	178.4 (4)	C13—P1—C19—C20	−82.7 (5)
C19—P1—C7—C8	65.5 (4)	C7—P1—C19—C20	28.6 (5)
Au—P1—C7—C8	−58.2 (4)	Au—P1—C19—C20	153.1 (4)
C12—C7—C8—C9	0.0 (8)	C24—C19—C20—C21	−1.0 (9)
P1—C7—C8—C9	178.1 (4)	P1—C19—C20—C21	180.0 (5)
C7—C8—C9—C10	0.6 (9)	C19—C20—C21—C22	0.8 (10)
C8—C9—C10—C11	−0.6 (10)	C20—C21—C22—C23	−1.2 (11)
C9—C10—C11—C12	0.0 (9)	C21—C22—C23—C24	1.7 (12)
C8—C7—C12—C11	−0.5 (8)	C22—C23—C24—C19	−1.8 (11)
P1—C7—C12—C11	−178.6 (4)	C20—C19—C24—C23	1.5 (10)
C10—C11—C12—C7	0.5 (8)	P1—C19—C24—C23	−179.4 (5)
C19—P1—C13—C14	−159.3 (4)		

### Hydrogen-bond geometry (Å, °)

**Table:**

*D*—H···*A*	*D*—H	H···*A*	*D*···*A*	*D*—H···*A*
C8—H8···N1^i^	0.94	2.54	3.433 (9)	159

Symmetry codes: (i) −*x*, −*y*, −*z*.
